# Public responses to the sharing and linkage of health data for research purposes: a systematic review and thematic synthesis of qualitative studies

**DOI:** 10.1186/s12910-016-0153-x

**Published:** 2016-11-10

**Authors:** Mhairi Aitken, Jenna de St. Jorre, Claudia Pagliari, Ruth Jepson, Sarah Cunningham-Burley

**Affiliations:** 1 Usher Institute of Population Health Sciences and Informatics, University of Edinburgh, Medical School, Teviot Place, Edinburgh, EH8 9AG UK; 2 The Scottish Collaboration for Public Health Research (SCPHRP), 20 West Richmond Street, Edinburgh, EH8 9DX UK

**Keywords:** Data linkage, Data sharing, Public engagement, Health informatics

## Abstract

**Background:**

The past 10 years have witnessed a significant growth in sharing of health data for secondary uses. Alongside this there has been growing interest in the public acceptability of data sharing and data linkage practices. Public acceptance is recognised as crucial for ensuring the legitimacy of current practices and systems of governance. Given the growing international interest in this area this systematic review and thematic synthesis represents a timely review of current evidence. It highlights the key factors influencing public responses as well as important areas for further research.

**Methods:**

This paper reports a systematic review and thematic synthesis of qualitative studies examining public attitudes towards the sharing or linkage of health data for research purposes. Twenty-five studies were included in the review. The included studies were conducted primarily in the UK and North America, with one study set in Japan, another in Sweden and one in multiple countries. The included studies were conducted between 1999 and 2013 (eight studies selected for inclusion did not report data collection dates). The qualitative methods represented in the studies included focus groups, interviews, deliberative events, dialogue workshops and asynchronous online interviews.

**Results:**

Key themes identified across the corpus of studies related to the conditions necessary for public support/acceptability, areas of public concern and implications for future research. The results identify a growing body of evidence pointing towards widespread general—though conditional—support for data linkage and data sharing for research purposes. Whilst a variety of concerns were raised (e.g. relating to confidentiality, individuals’ control over their data, uses and abuses of data and potential harms arising) in cases where participants perceived there to be actual or potential public benefits from research and had trust in the individuals or organisations conducting and/or overseeing data linkage/sharing, they were generally supportive. The studies also find current low levels of awareness about existing practices and uses of data.

**Conclusions:**

Whilst the results indicate widespread (conditional) public support for data sharing and linkage for research purposes, a range of concerns exist. In order to ensure public support for future research uses of data greater awareness raising combined with opportunities for public engagement and deliberation are needed. This will be essential for ensuring the legitimacy of future health informatics research and avoiding further public controversy.

## Background

Since the publication of the World Medical Association’s Declaration on Ethical Considerations regarding Health Databases in 2002, which stated that “databases are valuable sources of information” for health research, quality assurance and risk management [1] there has been steady and significant growth in the sharing of health data for ‘secondary uses’. The Medical Research Council (MRC) and Wellcome Trust ([2], p.6) note that “recent years have brought many calls for the optimisation of data sharing for research, with the intention of deriving maximal societal benefit”.

Recently this commitment to expanding research uses of data has led to growing interest in the public acceptability of data sharing and data linkage practices (e.g. [3]). This relates, in part, to the recognition of the importance of ensuring that data uses align with public interests or preferences. Recent highly publicised controversies (for example relating to care.data in England) have drawn attention to the importance of ensuring public support for the ways that data are used. Thus, there is increasing attention to public acceptability of secondary uses of data and to ensuring that these uses are understood and supported by the wider public (from whom the data originate). This may be crucial for ensuring the legitimacy of current practices and systems of governance. As Bradwell and Gallagher [4] have suggested; “personal information use needs to be far more democratic, open and transparent” and this means “giving people the opportunity to negotiate how others use their personal information in the various and many contexts in which this happens” (pp:18–19).

Previously it was noted that the literature in this area was dominated by practitioner perspectives and public views were underrepresented or underreported [5]. However, over the last decade there has been a steady increase in the number of studies exploring public attitudes or acceptability of secondary uses of data. Such studies have been conducted in a range of contexts and in relation to various research practices. Qualitative studies in the field of medical and healthcare research have, historically, tended to receive less attention than quantitative studies. However, despite qualitative studies usually being based on small sample sizes that prohibit claims to being statistically representative [6, 7], they can provide rich insights and a deeper understanding of the complexities or nuances of public opinions and experiences. They also allow for public views to be interpreted in a way that can effectively inform policy and practice issues [8]. Recently, reports discussing public views toward data sharing or data linkage for research purposes have principally used qualitative methods [3, 9, 10], exemplifying the value of such approaches for exploring the challenges and complexities of this topic.

Data-sharing and data-linkage refer to two distinct processes which are used in different ways. Data-sharing involves information moving from one organisation or department to another, whereas data-linkage is defined as: “the bringing together from two or more different sources, data that relate to the same individual, family, place or event” [11]. Increasing amounts of health research are conducted through data-linkage, for example health related records have been linked with population registries [12], alcohol and drugs services [11], genealogical registries [11], the census [13, 14]), the education system [15] and the prison service [16]. Such linkages have enabled, among other things, examination of relationships between social factors and health or access to health services.

This paper reports the results of a systematic review and thematic synthesis of qualitative studies which have explored public attitudes to data-sharing or data-linkage for research purposes. The study aimed to address the following research question:

What are the key issues of public responses in data-sharing and data linkage for research, and how have these been characterised?

This paper reports key themes that have emerged through this thematic synthesis and discusses their relevance for current debates around secondary uses of data for health research. Given the growing international interest in this area this represents a timely review of current evidence. It highlights the key factors influencing public responses and in doing so identifies particular topics of salience which it will be important to examine further.

Throughout this paper the terms ‘review’, ‘researcher(s)’, ‘participant(s)’ and ‘author(s)’ will be used to refer to this systematic review, the authors of the included studies, the research participants of each study and the authors of this paper, respectively.

## Methods

### Search strategy and inclusion criteria

A systematic literature search was conducted of five electronic databases (CINAHL Plus, EMBASE, Medline, Scopus and Web of Science) on 4 April 2014. Table 1 displays the key search terms that were tailored for all databases using both free-text terms and subject headings where possible (see Appendix 1 for an adapted search strategy for Medline). In addition, searches were conducted through Google Scholar and Open Grey as well as scanning references of included papers and contacting experts for a more inclusive result. There were no limitations on publication dates, languages or geographical locations.

**Table 1:** Key search terms

(lay OR patient* OR public OR citizen$1) AND (attitude* OR view$1 OR perspective* OR opinion$1) AND (data OR record$1) AND (share$ OR sharing OR link$2 OR linkage) AND Research AND (access OR purpose$1) AND (qualitative OR ethnograph* OR “grounded theory” OR “in depth interview$1” OR “structured interview$1” OR “focus group$1” OR “case study” OR “case studies” OR “case series” OR “citizen$2 juries” OR vignette* OR observation*)	
**□** Asterisks (“*”) are used as a wildcard to allow for any given search term to be truncated or remain the same.	
**□** Dollar signs (“$”) followed by a number refer to the number of additional characters allowed for each selected term.	

**Fig. 1:**
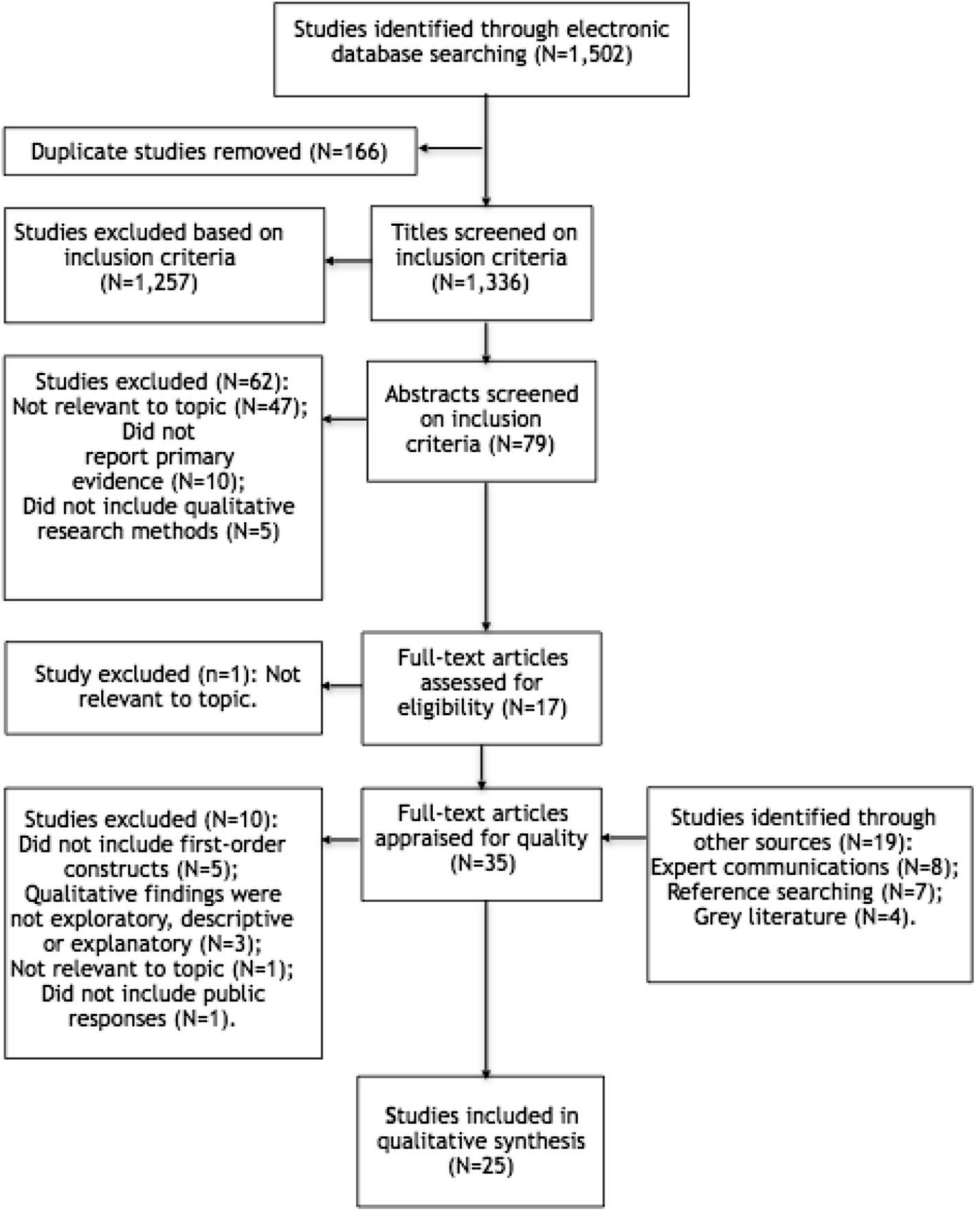
Selection process based on PRISMA flow diagram

The initial database searches revealed 1502 papers. Two authors (M.A. and J.S.J.) separately screened titles and abstracts and read eligible full texts before reconvening to discuss their results and resolve any discrepancies. Figure 1 shows the search and selection outcomes for each stage of the process. An additional 19 papers were identified through other sources (hand-searching references, expert communications and grey-literature searches). Papers were included if they met all inclusion criteria (see Table 2).

**Table 2:** Inclusion and exclusion criteria

	Inclusion Criteria	Exclusion Criteria
Study Design	All studies based on primary evidence. All studies reporting qualitative research relevant to the phenomenon [37] including, but not limited to: focus groups, in-depth interviews, case studies, vignettes, citizens’ juries, ethnographic observation, field notes. Mixed methods studies with a qualitative component that applied qualitative methods to collect and synthesise the research were included if the findings were presented separately from the quantitative data. All studies that included first-order constructs directly reporting participants’ responses, such as quotations [19]. All qualitative studies that provided exploratory, descriptive and/or explanatory findings [38].	All studies based on secondary evidence. All studies that were solely based on quantitative research. All studies that did not include first-order constructs directly reporting participants’ responses, such as quotations [19].
Study Population	All studies researching public (including patient/lay) perspectives. Studies involving public and expert opinions were included if public and expert responses were reported separately.	All studies that did not report public perspectives.
Research Topic	All studies that discussed the sharing and linkage of data in research.	All studies that did not discuss the sharing and linkage of data in research.

### Quality appraisal and data extraction

Each included study was individually appraised and data were extracted by the same two authors. Table 3 displays the main characteristics of each study including the study aim, date of data collection, setting, sample characteristics, sampling and method of data collection. The Critical Appraisal Skills Programme [17] checklist was used to critically assess the qualitative research. It was agreed that all studies were of sufficient quality to be included in the study. The CASP checklist represented a valuable tool for facilitating critical reflection on each of the studies.

**Table 3:** Study characteristics

No.	Reference	Study aim	Date of data collection	Setting	Sample (N, gender, age)	Sampling	Method of data collection
1	Aitken, M., 2011, Linking Social Care, Housing and Health Data: Social care clients’ and patients’ views Scottish Government Social Research	To explore the perspectives of social care clients, patients and carers in regards to linking social care, housing support and health data in statistical research and their views of possible impacts on privacy.	May 2011 (comparative study with data collected between October 2010 and February 2011).	North Ayrshire, Edinburgh and Tayside; Scotland	Patients, service users, carers and the wider public (*N* = 20): 85 % female and 57 % over the age of 60 (70–79 years = 14 %; 60–69 years = 43 %; 50–59 years =14 %; 40–49 years = 5 %; no reported age = 24 %). Over half (60 %) of the sample stated they suffered from a health problem or disability for at least 12 months.	-	A series of consumer panels (*N* = 3) involving semi-structured group discussions
2	Asai, A., Ohnishi, M., Nishigaki, E., Sekimoto, M., Fukuhara, S., & Fukui, T, 2002, ‘Attitudes of the Japanese public and doctors towards use of archived information and samples without informed consent: Preliminary findings based on focus group interviews’ BMC Medical Ethics 3(1): 1	To explore lay attitudes toward the use of archived health materials and to compare their views with those of physicians involved in medical research.	November 2000	Osaka, Japan	Men of the general public (*N* = 7), women of the general public (*N* = 7), and male physicians (*N* = 7), all between 35 and 55 years old (members of the general public were married with children)	-	Focus-group interviews (*n* = 3), one for each sample group
3	Bond, C. S., Ahmed, O. H., Hind, M., Thomas, B., & Hewitt-Taylor, J., 2013, ‘The conceptual and practical ethical dilemmas of using health discussion board posts as research data’ Journal of Medical Internet Research 15(6)	To explore the perspectives of contributors to online diabetes discussion boards to better understand their opinions towards how their shared information should be used by health researchers.	April to May 2012	World Wide Web	People living with diabetes who use online discussion boards (*N* = 26): male (*N* = 12), female (*N* = 9), unknown (*N* = 6).	-	Online semi-structured asynchronous email interviews
4	Damschroder, L. J., Pritts, J. L., Neblo, M. A., Kalarickal, R. J., Creswell, J. W., & Hayward, R. A., 2007, ‘Patients, privacy and trust: Patients’ willingness to allow researchers to access their medical records’ Social Science & Medicine 64(1):223-235	To better understand why there was wide variation in consent process recommendations despite a high inclination to share medical records for research.	November 2003 to June 2004	Four diverse Veteran Affairs (VA) facilities, USA	Patients from 4 Veterans Affairs facilities (*N* = 217); 95 % male; 37 % minorities; average age was 65 years.	Random and purposive sampling, stratifying for clinic visits, age and race/ethnicity	Mixed methods: Deliberation sessions (*N* = 39), preceded by a baseline-phone-administered survey and proceeded by another phone administered survey 4–6 weeks after the deliberation session.
5	Davidson, S., McLean, C., Cunningham-Burley, S. & Pagliari, C. 2012, Public Acceptability of Cross-Sectoral Data Linkage: Deliberative research findings Scottish Government Social Research	To explore public opinion on the acceptability of linking personal data for statistical and research purposes, by identifying particular sensitivities and possible barriers to public confidence and exploring ways to overcome concerns.	26 May to 9 June 2012	Stirling, Inverness and Glasgow; Scotland	Members of the public (*N* = 73): Male (*N* = 35); female (*N* = 38). Age groups: 18–34 years (*N* = 25), 35–49 years (*N* = 23) and 50+ years (*N* = 25).	Quota sampling	Series of public deliberative event: Half-day workshops were held in each location (*N* = 3).
6	Davidson, S., McLean, C., Treanor, S., Aitken, M., Cunningham-Burley, S., Laurie, G., Pagliari, C & Sethi, N., 2013, Public Acceptability of Data Sharing Between the Public, Private and Third Sectors for Research Purposes (Social Research series). Scottish Government	To build on previous research, existing literature and practical examples to better understand the issues related to data sharing across the public, private and third sectors for statistical and research purposes.	Oban - 29 June 2013; Aberdeen - 6 July 2013; Glasgow - 13 July 2013; Galashiels - 20 July 2013; and Edinburgh - 3 August 2013.	Oban, Aberdeen, Glasgow, Galashiels and Edinburgh; Scotland	Participants attending the general public workshops (*N* = 105): Men (*N* = 52) and women (*N* = 53). Age bands: 18–34 years (*N* = 28); 35–49 years (*N* = 32); and 50+ years (*N* = 35). Pregnant/child < 1 year old (*N* = 13). Participants attending the LGBT workshop (*N* = 12): Men (*N* = 6) and women (*N* = 6). Age bands: 18–34 years (*N* = 5); 35–49 years (*N* = 5); 50+ years (*N* = 2).	Quota sampling	Primary and secondary research methods: Literature reviews (*N* = 2) and a series of deliberative events (4 half-day events with members of the general public and a separate, smaller scale event of LGBT people).
7	Grant, A., Ure, J., Nicolson, D. J., Hanley, J., Sheikh, A., McKinstry, B., & Sullivan, F., 2013, ‘Acceptability and perceived barriers and facilitators to creating a national research register to enable 'direct to patient' enrolment into research: the Scottish Health Research Register (SHARE)’ BMC Health Services Research 13(1): 422	To explore patients’, clinicians’, healthcare management staff and researchers’ acceptability and feasibility of the national research register model to better understand their perspectives on the main facilitators and barriers to engagement in research.	Between February and June 2011	Local health centres in Tayside and Lothian areas in Scotland	Patients (*N* = 37); health service researchers (*N* = 10); general practitioners and practice managers (*N* = 17).	Purposive and convenience sampling	Focus groups with patients (*N* = 7); additional focus group with health service researchers (*N* = 1); semi-structured interviews with health service staff (*N* = 17).
8	Haddow, G., Bruce, A., Sathanandam, S., & Wyatt, J. C., 2011, ‘‘Nothing is really safe’: a focus group study on the processes of anonymizing and sharing of health data for research purposes’ Journal of Evaluation in Clinical Practice 17(6): 1140-1146	To explore lay opinions about the anonymisation and data-sharing processes to Scotland’s ‘warehouse’ model and to discuss whether consent is necessary.	May and June 2009	North East of Scotland	Lay participants from the Public Partnership Group (*N* = 19): Males (*N* = 6); females (*N* = 12); 1 participant did not reveal their gender. Ages 75 and over (*N* = 3); ages 60 to 74 (*N* = 15); ages 60 and under (*N* = 1). Participants with a chronic health condition (*N* = 13, 68 %).	-	Focus groups (*N* = 3).
9	Haga, S. B., & O’Daniel, J., 2011, ‘Public perspectives regarding data-sharing practices in genomics research’ Public Health Genomics 14(6): 319	To explore public attitudes, predominantly of African Americans, toward data sharing in genetic and/or genomic research and the possible impact of said practices on research involvement.	February 2008 to February 2009	Various locations across Durham, North Carolina, USA	Total participants (*N* = 100): 73 % female; 76 % African-American; median age of 40–49 years; 26 % had some college education but no degree.	-	Mixed methods: Focus groups (*N* = 10) ending with a survey.
10	Hill, E. M., Turner, E. L., Martin, R. M., & Donovan, J. L., 2013, ‘“Let’s get the best quality research we can”: public awareness and acceptance of consent to use existing data in health research: a systematic review and qualitative study’ BMC Medical Research Methodology 13(1): 72	To determine the varying opinions of the public regarding the use of existing medical data for research and to explore their views on consent to a secondary review of medical records for research purposes.	-	Dorset, England	Older male participants (*N* = 19): Ages 54 to 69 years; mean age 61 years; White British (*N* = 18) and White Other (*N* = 1).	Random sampling	Primary and secondary research methods: Systematic review (*N* = 1) and focus groups (*N* = 3).
11	Ipsos MORI, 2014, Dialogue on Data: Exploring the public’s views on using linked administrative data for research purposes HYPERLINK "https://www.ipsos-mori.com/DownloadPublication/1657_sri-dialogue-on-data-2014-census.pdf" www.ipsos-mori.com	To explore public views of the use of government administrative data in social research and to determine what kind of procedures, concepts and language are needed to be applied to build public confidence in the safety and security of the linking of their public records.	October to November 2013	England (London, King’s Lynn and Manchester), Wales (Cardiff and Wrexham), Scotland (Stirling), Northern Ireland (Belfast).	Public members (*N* = 129) and experts (*N* = 20).	Quota sampling	Seven sets of reconvened public dialogue workshops (*N* = 14 in total)
12	McGuire, A. L., Hamilton, J. A., Lunstroth, R., McCullough, L. B., & Goldman, A., 2008, ‘DNA Data Sharing: research participants' perspectives’ Genetics in Medicine 10(1): 46-53	To explore research participants’ attitudes and judgments toward data release and their preferences for differing levels of control over decision-making through three alternative types of consent (traditional, binary and tiered).	-	USA	Patients and controls (*N* = 15) from a genetic study on epilepsy. Age ranges of 18–70 years; females (*N* = 11); males (*N* = 4). Age bands 46–55 years and 56–65 years had the most participants for both focus-group sessions. Most participants had prior experience with medical research (*N* = 13).	-	Focus-group sessions (*N* = 3) containing both cases and control.
13	Melas, P. A., Sjöholm, L. K., Forsner, T., Edhborg, M., Juth, N., Forsell, Y., & Lavebratt, C., 2010, ‘Examining the public refusal to consent to DNA biobanking: empirical data from a Swedish population-based study’ Journal of Medical Ethics 36(2): 93-98	To empirically explore the motivations for not consenting to DNA biobanking in a population-based study and to examine the implications.	A longitudinal epidemiological project (PART) ongoing since 1998. The DNA-collection wave took place from 2006 to 2007.	Stockholm, Sweden	Participants from a longitudinal epidemiological project that had declined to contribute saliva samples for the DNA-collection wave. Participants interviewed (*n* = 23). Participants in ‘interview subgroup 1’ (*N* = 15): men (*N* = 8), mean age 48 years, range 33–61 years; women (*N* = 7), mean age 47 years, range 38–69 years. Participants in ‘interview subgroup II’ (*N* = 8): men (*N* = 4), mean age 49.7 years, range 34–68 years; women (*N* = 4), mean age 48.7 years, range 38–69 years. Individuals completing the non-participation questionnaire (*N* = 903).	-	Mixed methods: longitudinal population-based study (PART) of structured questionnaires and semi-structured interviews (*N* = 23).
14	MRC & Ipsos MORI, 2007, The Use of Personal Health Information in Medical Research: General Public Consultation - Final Report HYPERLINK "http://www.mrc.ac.uk" www.mrc.ac.uk	To identify public concerns and misconceptions in relation to the secondary use of personal health information for medical research.	29 July 2006 - public workshops in London and Cardiff; 5 August 2006 - general workshop in Edinburgh; 14 to 18 September 2006 - large-scale quantitative survey	London, Cardiff and Edinburgh; UK	Workshop participants reflecting the general public (*N* = 63). Interviews with disabled people and people with long-term/chronic conditions or their carers (*N* = 6). A nationally representative sample completed the survey (*N* = 2106).	Quota sampling	Mixed methods: general public workshops (*N* = 3), in-depth interviews over the telephone (*N* = 6) and a large-scale quantitative survey.
15	Nair, K., Willison, D., Holbrook, A., & Keshavjee, K., 2004, ‘Patients' consent preferences regarding the use of their health information for research purposes: a qualitative study’ Journal of Health Services Research & Policy 9(1): 22-27	To investigate the consent preferences of patients whose de-identified health data are presently being used in research.	March 1999 to May 2000	Hamilton area, Ontario, Canada	Patients (*N* = 17): Male (*N* = 6); female (*N* = 11); mean age was 52 years. Patients had been with their physician for an average of 10 years. The median number of physician visits were 4/year.	Convenience sampling	Individual semi-structured interviews (*N* = 17).
16	O’Kane, A. A., Mentis, H. M., & Thereska, E., 2013, ‘Non-static nature of patient consent: shifting privacy perspectives in health information sharing’ Proceedings of the 2013 Conference on Computer Supported Cooperative Work (CSCW-2013) 553–562. New York: ACM	To explore the views of chronically ill patients and their carers towards the use of their personal medical information and how it has impacted their perspectives of sharing their records with healthcare providers and secondary-use organisations.	-	Eastern England and the Greater London area; England	Diabetic patients (*N* = 27) diagnosed with Type I or Type II diabetes who were in charge of their own self-care and diabetic specialists (*N* = 6): Male (*N* = 15); female (*N* = 12); 18–73 years age ranges; 2–32 years diagnosis ranges; patients who had Type I diabetes (*N* = 12); patients who had Type II diabetes (*N* = 15). Diabetes health specialists (*N* = 6).	Theoretical, non-probabilistic sampling (Convenience sampling to interview the diabetes specialists)	Individual interviews (patients and specialists) and a group interview with 12 patients.
17	Robling, M. R., Hood, K., Houston, H., Pill, R., Fay, J., & Evans, H. M., 2004, ‘Public attitudes towards the use of primary care patient record data in medical research without consent: a qualitative study’ Journal of Medical Ethics 30(1): 104-109	To explore public and lay opinions about the use of primary care records for research when patient consent has not been sought.	-	South Wales	Members of the public participated in the focus groups (*N* = 49): participants older than 50 (*N* = 32; 71 %); participants in a non-manual social class (*N* = 36). Non-medical members of local community health councils participated in the key informant interviews (*N* = 4).	Stratification of groups by gender, geographical setting and level of deprivation.	Focus-group meetings (*N* = 8) and semi-structured key informant interviews (*N* = 4).
18	Saxena, N., & Canadian Policy Research Networks. Public Involvement Network, 2006, Understanding Canadians' attitudes and expectations: citizens' dialogue on privacy and the use of personal information for health research in Canada CPRN= RCRPP	To explore Canadian’s views about personal privacy and the use of personal information for health research purposes.	April to May 2005	Hamilton, Halifax, Vancouver, Montreal, Toronto; Canada	Members of the public (*N* = 98): male (*N* = 40); female (*N* = 58). Age ranges: 20–39 years (*N* = 37), 40–59 years (*N* = 35), 60+ years (*N* = 26). Women were over-represented at the dialogue in comparison to the general population (59 % to 51 %, respectively).	Random-digit dialling	One-day citizen dialogue sessions in English (*N* = 5) and French (*N* = 2).
19	Spruill, I. J., Gibbs, Y. C., Laken, M., & Williams, T., 2014, ‘Perceptions toward establishing a biobank and clinical data warehouse: Voices from the community’ Clinical Nursing Studies 2(3): 97	To assess community opinions of the proposed Biobank practices and policies for storing biomaterial for future research, and to determine the best practice for educating the public regarding the biobank and Clinical Data Warehouse (CDW).	-	Charleston, Beaufort, Berkeley, Dorchester, Georgetown and Horry; South Carolina, USA	Total participants (*N* = 67): key informants (*N* = 10) and general community members (*N* = 57). Focus-group participants were mostly African-American females from Georgetown and Beaufort counties. The cognitive-interview sample included key informants from the faith/scientific community, medical staff and formal/informal community leaders.	N/a	Focus groups of general community members (*N* = 7) and individual key informant interviews (*N* = 10).
20	Stone, M. A., Redsell, S. A., Ling, J. T., & Hay, A. D., 2005, ‘Sharing patient data: competing demands of privacy, trust and research in primary care’ British Journal of General Practice 55(519): 783-789	To explore the knowledge and attitudes of patients and primary healthcare staff regarding the sharing of data held in medical records, particularly for research and how this may affect trust between patients and healthcare professionals.	-	Five general practices in Leicestershire, England.	Patients (*N* = 20) and healthcare professionals/managers (*N* = 15).	Purposive and quota sampling	Semi-structured interviews
21	TNS 2012, Open Data Dialogue: Final Report	To examine how open data principles and policies in research should be developed and implemented.	Late February to early March 2012	Swindon, Oldham and London; England	Members of the public (*N* = 40). The same participants engaged in both Wave 1 and Wave 2.	-	Primary and secondary research methods: Literature and policy review (*N* = 1); public-dialogue workshops (*N* = 2); stakeholders mapping and scoping workshop (*N* = 1); and producing a report for dissemination (*N* = 1).
22	Trinidad, S. B., Fullerton, S. M., Bares, J. M., Jarvik, G. P., Larson, E. B., & Burke, W.,2010, ‘Genomic research and wide data sharing: views of prospective participants’ Genetics in Medicine 12(8): 486-495	To explore the views of current and possible future research participants regarding genome-wide association studies (GWAS) and repository-based research.	March to August 2008	Seattle metropolitan areas, Washington, USA.	Current research participants in the Adults Changes in Thought (ACT) Study; ACT surrogate decision-makers; and three age-defined cohorts of Group Health members not in the ACT Study of 18–34 years, 35–50 years and 50+ years (*N* = 79). Mean age was 56.4 years; age range was 18–89 years; 48 % female.	Random sampling	Series of focus-group discussions (*N* = 10): Two sessions were held for each of the 5-selected population groups.
23	Trinidad, S. B., Fullerton, S. M., Bares, J. M., Jarvik, G. P., Larson, E. B., & Burke, W., 2012, ‘Informed consent in genome-scale research: what do prospective participants think?’ AJOB primary research 3(3): 3-11	To explore the views of research participants and members of the general public toward informed consent and issues involving genome-wide association studies (GWAS) and other similar kinds of genomic research.	March to August 2008	Seattle, Washington, USA	Members of the Group Health Cooperative (*N* = 45): Mean age was 45 years; 40 % were female. Two separate sessions were held with members aged 18 to 34 years, members aged 35 to 50 years and members older than 50 years.	Random sampling	Focus groups (*N* = 6)
24	Weitzman, E. R., Kaci, L., & Mandl, K. D., 2010, ‘Sharing medical data for health research: the early personal health record experience’ Journal of Medical Internet Research 12(2)	To understand consumer willingness to share data from Personally Controlled Health Records (PCHR) for health research purposes and to explore the conditions and contexts that encourage willingness to share.	-	Urban area within the northeastern region of the USA.	Early adopter sample of PCHR users completed the surveys (*N* = 151). PCHR usability testers (*N* = 13) were recruited for the structured interviews and community members (*N* = 17) participated in the focus groups. Average age in the usability test group was 45 years, reflecting an employee and student population. Average age across focus-group participants was 71 years, drawn from a retiree and health advocacy mailing list. Females outnumbered males in all groups.	-	Mixed methods: Surveys, interviews and focus groups.
25	Willison, D. J., Keshavjee, K., Nair, K., Goldsmith, C., & Holbrook, A. M., 2003, ‘Patients' consent preferences for research uses of information in electronic medical records: interview and survey data’ BMJ 326(7385): 373	To determine patients’ preferred method of consent for the use of information from electronic medical records for research purposes.	-	Southern Ontario, Canada	Patients of doctors in the COMPETE study (*N* = 123). Participants interviewed (*N* = 17): Male (*N* = 7); female (*N* = 11). Patients who completed a survey (*N* = 106).	-	Mixed methods: Semi-structured interviews and a structured fixed-response survey

### Synthesis

A thematic synthesis approach was adopted using Thomas and Harden’s [18] three-step technique: Free line-by-line coding of the included studies, the emergence of descriptive themes from the codes and the development of analytical themes. Independently, M.A. and J.S.J. coded the included studies using an inductive approach without *a priori* codes.

All authors met to discuss the codes/themes and to resolve any discrepancies. Three authors (R.J., C.P. and S.C.B.) were each assigned three articles (totalling nine) from the included studies to validate the findings of M.A. and J.S.J. At this stage ten further studies were excluded from the synthesis for not reporting participants’ verbatim views or first-order constructs (Britten et al. 2002); not reporting detailed qualitative findings (Sandelowski and Barroso 2003); not reporting findings relevant to the research topic; or for not including public responses. A list of descriptive themes (referred to as ‘sub’ themes) were agreed and organised by analytical (‘key’) themes. The key themes were identified and interpreted in relation to the research question (see Table 4). From the included studies, M.A. and J.S.J. extracted first- and second-order constructs (the latter being the original researchers’ interpretations of the participants’ constructs) including any *reciprocal* or *refutational translations* (comparable or opposing views) [19].

**Table 4:** Key themes and sub-themes

Key themes	Sub themes	Studies
Widespread Conditional Support	General widespread, yet conditional, support for uses of data in health research.	3, 7, 8, 10, 19, 21, 22, 25
	Health research or more general research is typically “in the public interest” or will benefit “the greater good”.	2, 3, 4, 7, 8, 9, 10, 12, 13, 14, 15, 18, 19, 20, 21, 22, 25
	“Doesn’t this happen already?!”	7, 10, 11
	Research uses of data were considered to be in the public interest and should therefore be used, not wasted.	1, 2, 6, 19, 21, 22
Conditions for Support	Assurances of individuals’ confidentiality are crucial for public support.	2, 3, 4, 5, 6, 9, 11, 14, 17, 18, 21, 22, 24
	Public preferences for data to be anonymous.	1, 3, 5, 6, 9, 11, 14,15, 17, 20, 21, 22
	If the data is anonymous “What does it matter?!”	6
	Anonymisation is not a panacea.	8, 17
	Anonymisation is imperfect.	1, 5, 8, 11, 14
	Participants made a distinction between “plain stats” and more detailed qualitative information.	1, 3, 11, 14
	Assurances of safeguards in place to protect against misuse or abuse of data were considered important for ensuring public support and/or acceptability.	1, 7, 8, 10, 11, 14, 18, 19, 21, 22, 24
	Public preferences for strong accountability mechanisms to be in place.	4, 5, 12, 14, 18, 22
	Low awareness of current research practices.	8, 10, 11, 14, 15, 17, 19, 20, 21, 25
	Low public awareness of current governance or ethics processes.	7, 10, 19, 21
	Assurances of data security are important for public acceptability of research uses of data.	7, 8, 10, 11, 24
	Concerns about data security were widely identified.	1, 5, 6, 8, 11, 14, 20, 22, 24
	Concerns about data security related to the fallibility of IT systems to protect against breaches.	5, 8, 11
	Concerns about data security related to human error were widely called upon.	5, 6, 8, 11, 23
	Breaches of security were regarded as always being possible, yet security risks were sometimes said to be tolerated or accepted where individuals valued the purpose and potential benefits of research.	8, 10, 22
	Public support was conditional if data would only be used for legitimate purposes.	8, 11, 19, 21, 22, 25
Benefits	Key condition for public support for research using individuals’ data was that such research must have public benefits.	1, 2, 4, 5, 6, 10, 11, 18, 21, 23, 25
	Perceived personal benefits, or personal relevance of research was reported to motivate participation in research.	7, 11, 13, 19, 23
	Concerns relating to personal privacy were balanced with recognition of the importance of societal benefits anticipated to come from research.	4, 5, 8, 9,14, 15, 17, 21, 22, 24
	Societal benefits prioritised *over* personal privacy.	21, 22
	Assurances that research would - or at least have the potential to - bring about public benefits were fundamental for ensuring public support or acceptance.	1, 2, 4, 5, 6, 10, 11, 18, 21, 23, 25
Control and Consent	Perceived autonomy, or individual control over how data is used, was found to be a key factor shaping public responses.	4, 12, 14, 18, 23, 25
	Members of the public value having control over their own data.	2, 12, 14, 18, 23, 25
	Participants explicitly referred to control over their own data in terms of individual or human rights.	4, 14, 15, 17, 25
	There was an evident link between levels of trust (in research organisations or data controllers) and desired level of individual control.	4, 25
	Individual control needs to be balanced with efficiency of research.	5, 10, 12, 18, 25
	Consent as a mechanism for facilitating individual control.	12, 15, 23, 25
	Varied views on consent and what form this should take.	2, 11, 12, 14, 20, 22, 23, 24
	Public preferences for explicit opt-in consent models.	14, 15, 19, 23
	Public acceptance of opt-out models in recognition of the challenges or practical limitations of opt-in.	10, 19
	Public preference for varied or flexible consent models which would enable individuals to set limits on their consent, or to indicate particular preferences or objections.	4, 5, 12, 18, 22, 23, 24
	Public objections to one-time consent models which would not allow individuals to review or change their consent preferences.	16, 23, 24
	Public opinions or preferences are not fixed but change and adapt in response to information, deliberation, events or circumstances.	11, 15, 16, 20, 21, 23, 25
	Consent was recognised to be problematic.	8, 10, 12, 17, 21, 22, 23
	Acknowledgment of the potential for selection bias or low participation rates if explicit opt-in consent is required.	10
	Consent regarded as important in relation to named or identifying data.	5, 20, 21
	Consent regarded as important in relation to qualitative information rather than “plain stats”.	3
	Consent regarded as important in relation to research using genetic data.	18, 19, 24
	Consent regarded as important in relation to where a commercial entity is involved in research.	18
	Consent was widely viewed to be important and in this regard, represented as an act of courtesy.	8, 17, 20, 23, 25
Uses and Abuses of Data	A key concern regarding research uses of data was the potential for data to be misused or abused.	4, 5, 8, 10, 11, 12, 13, 14, 21, 22
	Concerns about data being sold or passed on to third parties.	4, 8, 9, 11, 12, 14, 19, 21, 22, 23, 25
	Concern about data being used for political purposes.	5, 11, 21, 24
	Concerns about potential future uses of data.	5, 9, 11, 12, 13, 19, 21, 22, 23
	Concerns about potential “slippery slopes” as more information becomes accessible.	14
	Concerns about data being used for purposes other than those which were originally described.	17, 21, 23
	Concerns about the proliferation of data within modern societies and increasing surveillance through data collection - “Big Brother Society”.	5, 11, 13, 14, 22
	Concern related to the potential for stigma or discriminatory treatment to result from research which would label or categorise groups within society.	1, 5, 6, 8, 11,14,19
	Concerns relating to potential indirect negative impacts on individuals from participating in research (e.g. increased or denied insurance premiums due to information being accessed from medical records, etc.).	1, 5, 6, 9, 11,12, 17, 24
	Participants made differentiations between types of data and regarded some as more sensitive - and concerning - than others (e.g. mental health, sexual health, sexuality and religion).	6, 7, 11, 15, 16, 17, 20, 22, 23
Private Sector Involvement	Concern about private sector involvement in research using individuals’ data.	5, 6, 7, 8, 9, 10, 11, 12, 14, 17, 20, 22, 24
	Low levels of public trust in the private sector.	4, 5, 10, 11, 12, 14, 18, 21, 22, 23, 25
	Perceptions that private sector organisations are motivated by profit.	6, 10, 11, 20, 22
	Distinctions were made between research perceived to be “for profit” and research perceived to be “for the greater good”.	6, 7, 10, 11, 21, 22, 25
	Distinctions were made between “research purposes” and “commercial gain”.	3, 22
	Participants wanted assurances that public benefits would be prioritised over profit.	6, 10, 14, 18, 21, 25
	Participants wanted assurances that individuals’ privacy would be prioritised over profit.	18
	Participants wanted assurances that profits would be shared or reinvested so as to create public/societal benefits.	6
	Participants felt it was appropriate that private sector organisations pay for access to public sector data.	6, 11, 22
	Acceptance of private sector organisations paying for access to public sector data if the revenue generated is appropriately re-invested in the public sector.	17, 25
	Widespread concern about private sector involvement in research balanced by recognition that private sector involvement in research can be important or valuable.	8, 11, 21, 22
	Private sector involvement represented as a “necessary evil”.	6, 7, 8
	The private sector was not regarded as a homogenous entity, but rather distinctions were made between private sector organisations.	6, 8
	Private sector involvement was acceptable as long as commercial actors did not have access to data.	15
	Concerns about private sector organisations as funders of research and the implications this may have for the integrity or objectivity of the research.	5, 10, 15, 21, 25
	Ambivalent views on government research.	6, 11, 15
	Concern government access to data.	9, 11, 12, 22, 24
	High levels of trust in universities and academic researchers.	7, 11, 12, 20, 22
	Lack of trust in universities and academic researchers.	4
Trust and Transparency	Levels of trust individuals place in research organisations, oversight bodies or government informs their level of support for research uses of data.	2, 4, 6, 11, 14, 22
	Trust is essential for ensuring public acceptance and/or participation in research.	2, 4, 6, 8, 9, 11, 13, 14, 21
	Higher levels of trust in the public sector compared to the private sector, largely related to greater confidence in accountability and data protection mechanisms within the public sector.	6, 11, 21
	High levels of public trust in primary healthcare providers.	5, 7, 8, 14, 15, 17, 25
	Higher levels of trust in known or familiar individuals or organisations.	4, 17, 20, 22, 24, 25
	Preference that data-sharing and research uses of data to be overseen within, and governed by the public sector.	5, 6, 11
	Preference that such processes are overseen and controlled by healthcare professionals (e.g. known/familiar individuals).	5, 14, 15
	To oversee and govern data-sharing and research uses of data may be overly burdensome to healthcare professionals and take valuable time and resources away from the provision of healthcare.	17
	Participants request for more information about current research practices and uses of data.	2, 4, 5, 6, 7, 9, 10, 11, 12, 13, 14, 15, 19, 21, 23, 24
	Transparency as to how data is used for research purposes is considered crucial for building public trust, and consequentially securing public support.	12, 14, 18, 21
	The importance of awareness raising to build trust and public support is emphasised in certain studies.	6, 7, 11, 14, 15, 18, 19
	There is public interest and enthusiasm for more meaningful forms of public engagement/involvement.	5, 6, 11
	Public engagement/involvement is essential for ensuring accountability.	5, 6, 15

While three authors (M.A., S.C.B. and C.P.) work directly in the field of public engagement regarding health informatics research, the remaining two authors (J.S.J. and R.J.) were not previously familiar with the literature or debates in this area. The involvement of authors without prior understandings or perspectives on the literature was valuable for ensuring an inductive approach. The authors discussed and deliberated the coding and analysis to ensure that the findings emerged from the included studies rather than being shaped by or confirming the expectations of authors who are actively engaged in this subject matter.

## Results

### Included Studies

A total of 1521 studies were identified from the systematic searches. From these, 25 studies were included in the review. The research was conducted primarily in the UK (five studies in Scotland, four in England, one in Wales and two across the UK) and in North America (seven studies in the USA and three in Canada) with one study set in Japan, another in Sweden and one worldwide. Data was collected from 1999 to 2013, though eight studies did not report data collection dates. The research participants included patients, service users, carers, surrogate decision-makers, lay persons and the general public ranging from 18 years of age to over 75 years. Six studies reported expert opinions from healthcare professionals, managers, health service staff and diabetes specialists in addition to the views of members of the wider public or patient groups. The qualitative methods of data collection included focus groups, interviews, deliberative events, dialogue workshops and asynchronous online interviews. Six studies included mixed methods using surveys or structured questionnaires. Additionally, three studies reported both primary and secondary research including a literature or policy review or systematic review.

Seven key themes were identified across the included studies: Widespread Conditional Support; Conditions for Support; Benefits; Control and Consent; Uses and Abuses of Data; Private Sector Involvement; and Trust and Transparency.

### Key Themes

#### Widespread Conditional Support

The included studies point to a clear trend that there was generally widespread—albeit conditional—support for uses of data in health research.^1^ This is typically expressed in relation to a view that health research—or research more broadly—is “in the public interest” or is expected to bring about benefits for “the greater good”.^2^ For example, one participant in study number 25 stated:

*“I think the medical research is going to be of general benefit to the general population and if my records can help; I think personally I would be quite willing to participate in any medical study that is of general benefit to the population. I just feel it is worthwhile to participate in these studies”* (Patient 4, Willison et al. 2003: 2)

Uses of data for health or medical research were often conceptualised in relation to the potential for discovery of new cures or treatments, or the improvement of healthcare services.

In several studies participants were reported as being surprised that data are not already more widely used, with questions being asked such as: *“Doesn’t this happen already?*!”.^3^ Many studies reported that participants considered research uses of data to be in the public interest and conversely that not using data was against the public interest since this was a resource which should be used, not wasted.^4^

Despite broad agreement that using health data for medical research is generally a good thing, across the studies it is evident that support for these data uses was never unconditional. A number of factors were identified as being important conditions for public support or acceptance.

#### Conditions for Support

In a large number of studies assurances of individuals’ confidentiality were reported as crucial for public support.^5^ Whilst confidentiality may be assured through various mechanisms, in the included studies this was largely associated with anonymisation of data. Public preferences for data to be anonymous were widely reported,^6^ for example in one study^7^ a participant stated:

*“[The public need] reassurance about anonymity because that’s what people worry about”*

Some individuals expressed a view that if the data are anonymous *“what does it matter?*!”.^8^ However, others noted that anonymisation is not an absolute guarantee of confidentiality 9 and in a number of studies participants recognised that the anonymisation process is imperfect and therefore did not fully or adequately protect individuals’ confidentiality.^10^ For example, it was said:

*“I think you’re right enough, it’s anonymised. But then if you’re dealing with particular areas, that again kind of cuts in to the anonymous factor, because if you’re looking at maybe, let’s say, a housing estate, so there’s only so many people, so it’s not…I don’t think there’s anything that’s truly anonymous; I think everything can be found out if you’ve got the wherewithal and the curiosity to find things out.”* 11

In a number of studies participants made a distinction between “plain stats” and more detailed qualitative information, with the former largely considered not to be concerning while the latter raised greater issues relating to confidentiality and privacy.^12^

Assurances of safeguards to protect against misuse or abuse of data were also widely considered important for ensuring public support/acceptability.^13^ Similarly, members of the public often expressed a preference for strong accountability mechanisms to be in place.^14^ However, there was generally found to be low public awareness of current research practices^15^ and in particular, of current governance or ethics processes.^16^ As such, in a number of studies it was reported that public acceptance increased after participants were informed about existing safeguards and governance mechanisms.

Assurances of data security were also found to be important for public acceptance of the use of health data in research^17^ and across the studies concerns about data security were widely identified.^18^ Such concerns related to the fallibility of IT systems to protect against breaches^19^ as well as to human error. Media reports of *“laptops left on trains”* or misplaced data were widely called upon to illustrate this latter point.^20^ However, in a number of studies it was reported that participants regarded breaches of security as always being possible, yet security risks were sometimes regarded as tolerable or acceptable where individuals valued the purpose and potential benefits of research.^21^

A further condition for public support was that data would only be used for legitimate purposes. Whilst the term “legitimate” was not always referred to explicitly, the included studies often suggested or concluded that the extent to which members of the public perceived uses of data to be legitimate influenced their responses or preferences.^22^ However, there were varying views on how, or by whom, legitimacy was to be defined.

#### Public Benefits

Another key condition for public support for research using individuals’ data was that such research must have public benefits.^23^ Whilst in some cases perceived personal benefits, or personal relevance of research was reported to motivate participation in research,^24^ benefits of research were largely conceptualised in terms of benefits to wider society, or *“the greater good”*. For example, study participants said:

“*…We wouldn’t have the national health service, we wouldn’t have drugs, we wouldn’t have anything, if it hadn’t have been for people being allowed to try things out in the past. So, I suppose, when you look at it like that, it is almost as if you have a moral duty to say, we have benefited, so why shouldn’t we contribute for [future generations?]”* 25

In many cases it was reported that concerns relating to personal privacy were balanced with recognition of the importance of societal benefits anticipated to come from research.^26^ Moreover, in two studies it was reported that some participants prioritised societal benefits *over* personal privacy.^27^

Assurances that research would bring about public benefits—or at least that it had the potential to bring about such benefits—were widely reported to be fundamental for ensuring public support or acceptance. If research is perceived to be focussed primarily at benefitting individual researchers (e.g. through advancing their careers or raising their profile), as having no clear practical application or “real-world” value, or as being conducted solely for profit this leads to concerns and opposition (or at least less support) for research uses of data.^28^

#### Control and Consent

Perceived autonomy, or individual control over how data is used, was found to be a key factor shaping public responses in a number of studies.^29^ It was reported that members of the public valued having control over their own data.^30^ Such control relates to what data are collected, who has access to this, how and with whom data is shared and for what purposes the data are used. In a number of studies participants explicitly referred to this control in terms of individual or human rights.^31^

Whilst perceived individual control clearly emerged as a key factor shaping public attitudes or acceptance of research uses of data, there was no clear consensus (across or within) the studies regarding what this control implied or necessitated. In some studies there was a clear link between levels of trust in research organisations or data controllers and desired level of individual control.^32^ This suggests that where individuals trust organisations handling their data they are less likely to favour more stringent forms of control. Conversely, when this trust is lacking individuals want to have greater control over their own data.

Preferences for control are also influenced by wider attitudes towards the value of research. In a number of studies it was found that, whilst individual control was highly valued, participants did not want this control to come at the cost of creating barriers to research. Thus it was often found that participants felt that individual control needs to be balanced with efficiency of research.^33^

Across the included studies control is largely discussed in relation to consent. There is evidence that members of the public also made this association and recognised consent as a mechanism for facilitating individual control.^34^ However, both between and within studies there were varied views on consent and what form this should take.^35^ Some studies indicated public preferences for explicit opt-in consent models,^36^ whilst an acceptance of opt-out models was also reported due to recognition of the challenges or practical limitations of opt-in.^37^ In a significant number of studies there was a clear preference for varied or flexible consent models which would enable individuals to set limits on their consent or to indicate particular preferences or objections.^38^ Similarly, some studies reported that participants objected to one-time consent models which would not allow individuals to review or change their consent preferences.^39^ This relates to the fact that public opinions or preferences are not fixed but change and adapt in response to information, deliberation, events or circumstances.^40^

Whilst consent was widely valued as a mechanism for facilitating individual control in many studies, it was also recognised to be problematic.^41^ In particular participants in the studies acknowledged the potential for selection bias or low participation rates if explicit opt-in consent is required. Such recognition led to some individuals becoming more inclined to support opt-out consent models or non-consented uses of data, however this trend was certainly not universal and others maintained that consent was always important.

The included studies highlight a number of areas where consent was regarded as particularly important, for example in relation to named or identifying data,^42^ qualitative information rather than “plain stats”,^43^ research using genetic data^44^ or where a commercial entity is involved in research.^45^

Where consent was acknowledged to be problematic and/or where individuals reported that they were largely unconcerned about research uses of data, consent was nevertheless widely viewed to be important. In a number of studies consent was in this regard represented as an act of courtesy with participants suggesting that they would be happy to allow their data to be used for research but that this should nonetheless not be used without their permission.^46^

#### Uses and Abuses of Data

A key area of concern regarding research uses of data related to the potential for data to be misused or abused.^47^ In some cases this related to concerns that individuals with access to data would use it maliciously or inappropriately, for example it was stated that:

*“there are some people, [that] regardless of the consequences will defy rules and regulations to justify their existence or to prove they can do it…”* (Damschroder et al. 2007: 231)

In other instances these concerns related to data being sold or passed on to third parties^48^ and used for commercial purposes, e.g.:

*“What I don’t like is any information being passed on to a third party, for promotion purposes. Say you’ve got a particular problem then it goes to a drugs supplier or something like that, that I would object to.”* (Participant 4, group 1, Hill et al. 2013: 6)

There was also concern about data being used for political purposes,^49^ e.g.:

*“If the Government are using the details for the benefit of society, I think that’s okay. But if the Government are using that data to then look at their next election campaign, or look at the independence campaign by looking at the demographics of a particular area, then I don’t know if that’s as acceptable. They’[d] simply be using our data for their own goals”* (Female, aged 18–34, Glasgow, Davidson et al. 2013).

Some participants in the studies expressed concerns about potential future uses of data.^50^ While current uses or research objectives may be regarded as acceptable participants expressed scepticism that such uses would remain clearly defined and limited. Some study participants were worried about potential *“slippery slopes”* with more and more information becoming accessible^51^ or with data being used for purposes other than those which were originally described.^52^

There were also concerns about the proliferation of data within modern societies and increasing surveillance through data collection. For some these concerns were expressed in relation to the creation of a “*Big Brother Society*”,^53^ e.g.:

*“You can’t move. You can’t do anything without somebody, somewhere knowing exactly what you’re up to”* (Female, depth interview, MRC & Ipsos-MORI 2007: 25)

A significant area of concern related to the potential outcomes or implications of research. In particular, study participants were concerned about the potential for stigma or discriminatory treatment to result from research which would label or categorise groups within society,^54^ e.g.:

*“I think research maybe tends to lump everybody together, and there must be individuals that would be totally different […] so it could lump everybody together and maybe that’s not what we want.”* (Tayside—Female4, Aitken 2011: 12)

*“Some universities might feel: ‘we don’t want to involve people from areas of deprivation, because we know they are less likely to finish their course and that’s bad for us, for our figures’”* (Male, oldest age group, Edinburgh) (Davidson et al. 2013: 70)

There were also concerns relating to potential indirect negative impacts on individuals from participating in research.^55^ For example, a frequent concern related to potential for insurance premiums to increase or be denied as a result of information being accessible from medical records. Additionally there was concern that employers may gain access to information which could be used to the detriment of individual employees. Participants were concerned that data which was shared could be accessed and used in ways which could be harmful for individuals, e.g.:

*“Money’s money but health is how you feel as well and if you’re being persecuted in a way because of that, it’s just going to make you worse”* (Female, depth interview, MRC & Ipsos-MORI 2007: 29).

*“People can judge them, so if they find out something about you because of your health you could be picked on”* (Female, depth interview, MRC & Ipsos-MORI 2007: 29).

Such concerns were particularly salient in relation to more sensitive forms of data. Across the studies it was reported that participants differentiated between types of data and regarded some as more sensitive—and concerning—than others.^56^ Examples of particularly sensitive forms of data include data relating to mental health, sexual health, sexuality and religion.

#### Private Sector Involvement

Across the studies there was significant concern about private sector involvement in research using individuals’ data.^57^ Such concerns largely related to two key factors: low levels of public trust in the private sector^58^ and a perception that private sector organisations are primarily—or solely—motivated by profit.^59^ Across the studies participants often made distinctions between research which was perceived to be *“for profit”* and research perceived to be *“for the greater good”.* 60 Similarly, distinctions were made between “research purposes” and “commercial gain”^61^ as if they were opposing motivations. As noted above, the creation of public benefits from research was widely regarded as an essential prerequisite for public support or acceptance. Therefore, where participants regarded research to be conducted for purposes other than creating public benefits this raised concerns.

However, such concerns did not necessarily mean outright opposition to private sector involvement in research. Profit-creation resulting from research was regarded as acceptable under certain conditions. Notably, the included studies indicated that participants wanted assurances that public benefits would be prioritised over profit,^62^ that individuals’ privacy would be prioritised over profit^63^ and that profits would be shared or reinvested so as to create public/societal benefits.^64^ Additionally, while there were concerns about individuals’ data being sold, studies which explored private sector access of public sector data found that participants often felt it was appropriate that private sector organisations pay for access to these data^65^ and that this would be regarded as acceptable on the condition that revenue generated is appropriately re-invested in the public sector.^66^

While there was widespread concern about private sector involvement in research this was often balanced by a recognition that private sector involvement in research can be important or valuable.^67^ In some cases private sector involvement was represented as a *“necessary evil*”,^68^ e.g.:

*“… the drug companies are just trying to make money, and yes of course they are, it’s all about money in the end of the day but if they don’t find the research for some of these the less interesting or less topical things then they, there will not be research into those things…we need to get funding from drug companies anyway, if they’re the ones with the money.”* (Female, patient focus group 3, PPG, Grant et al. 2013: 8).

Thus profit-creation was regarded by some study participants to act as an incentive for private sector organisations to conduct valuable research in the public interest.

Overall, the included studies demonstrate that members of the public hold nuanced and complex views regarding private sector involvement. It is noteworthy that the private sector was not regarded as a homogenous entity, but rather distinctions were made between private sector organisations.^69^ There was also acknowledgement of the different roles that private sector organisations can play in research. For example it was reported in one study that private sector involvement was acceptable as long as commercial actors did not have access to data.^70^ Other studies reported concerns about private sector organisations as funders of research and the implications this may have for the integrity or objectivity of the research.^71^

Whilst low trust in private sector actors is frequently reported, the included studies also demonstrate complex or ambivalent relationships of trust in actors from other sectors. For example, several studies identified ambivalent views on government research^72^ and concern about government access to data.^73^ Additionally, whilst some studies reported high levels of trust in universities and academic researchers^74^ one reported a lack of trust in university researchers.^75^ Thus relationships of trust are not straightforward and there does not appear to be a clear, or static hierarchy of trusted organisations/sectors.

#### Trust and Transparency

Trust is a key theme running through all of the included studies (both implicitly and explicitly). A number of studies indicated that the level of trust individuals place in research organisations, oversight bodies or government, informs their level of support for research uses of data.^76^ The included studies indicate that trust is essential for ensuring public acceptance and/or participation in research.^77^

As noted above, relationships of trust are nuanced and complex. However the included studies indicate generally higher levels of trust in the public sector compared with the private sector, largely related to greater confidence in accountability and data protection mechanisms within the public sector.^78^ There is also evidence of particularly high levels of public trust in primary healthcare providers.^79^ This reflects a trend of higher levels of trust in known or familiar individuals or organisations,^80^ which was exemplified in study participants’ confidence in particular healthcare professionals to make good judgements on access to patients’ data:

*“I know my physician well enough to have a good feel for the types of things he would be involved with”* (Patient 12, Nair et al. 2004: 25).

*“If you trust the doctor, I don’t think it would worry me how much [data] you needed, and I do trust the doctor”* (Patient 15, Nair et al. 2004: 25).

It also leads to individuals preferring to be contacted only by healthcare professionals, or known individuals:

*“I am happy to have personal contact with our hospital, GP or the health professionals who knows me, but I am not happy being contacted by a Pfizer company, or whatever”* (MRC & Ipsos-MORI 2007: 19).

Participants in the included studies often expressed a preference that data-sharing and research uses of data be overseen within, and governed by, the public sector.^81^ In some instances there was a preference for such processes to be overseen and controlled by healthcare professionals (e.g. known/familiar individuals).^82^ However, some study participants acknowledged that this may be overly burdensome and take valuable time and resources away from the provision of healthcare.^83^

The importance of relationships and familiarity to trust is indicative of a broader desire for greater transparency about research practices. The included studies overwhelmingly suggest an appetite among study participants for more information about current research practices and uses of data.^84^ Transparency about how data is used in research is considered crucial for building public trust, and thereby securing public support.^85^ Moreover, many of the included studies point towards the importance of awareness raising for building trust and public support.^86^

However, the included studies highlight that the public should not be conceived of as simply subjects of information provision relating to research uses of data. Rather, several studies indicate public interest and enthusiasm for more meaningful forms of public engagement/involvement.^87^ Such involvement was considered essential for ensuring accountability.^88^

#### Differences between studies

It is not possible to make clear or consistent comparisons between the findings of the included studies due to different social and cultural contexts. For example, in a Japanese study^89^ participants were reported to describe “unequal relationships” between patients and doctors with patients belonging to a “lower rank”. This may reflect (actual or perceived) traditional doctor-patient relations in Japan that are more hierarchical and paternalistic [20]. However, discussions of unequal relationships in other studies were not explicitly reported though some study participants may have implicitly referred to them. Diverse study populations also limit the findings from being comparable. These smaller populations include U.S. veterans reporting higher levels of trust and greater support for research by Veteran Affairs^90^; African Americans expressing lower willingness to engage in genetic/genomic research due to past abuses^91^; and LGBT participants in the U.K. concerned for the misuse of data, particularly identifiable data, that could lead to discriminating opinions and behaviour.^92^ These findings build on previous research reporting concerns over the underrepresentation of minority populations in research, such as African Americans [21–23] and LGBTs [24]. While these views may not be comparable to other contexts, they are indeed essential to understanding the needs of different social groups to better inform a wide variety of policies and practices. Despite variations in opinion, the overall views of these study populations were consistent with the general findings of the thematic synthesis.

A further limitation to the review was the underrepresentation of young people across the studies. Of the few studies that compared all age groups, the variations in opinion were detailed. Two studies reported that younger participants expressed greater concerns for privacy and a desire for control over research data.^93^ Another noted that some felt “anxious” about their data being held while others believed they had little control over their own information.^94^ In contrast, older participants were reported to favour less individual control^95^ or to be less worried about the possible loss of confidentiality.^96^ Previous research by Buckley et al. [25] equally commented on the lack of participation of younger people in their study. The few that responded, were more cautious about the use of their medical information compared to older participants. However, the researchers were wary of these results due to the unrepresentativeness of the sample. Additionally, there are some contradictory findings, for example, King et al. [26] found that younger participants and older respondents over the age of 60 were less concerned about the privacy of their health information compared to participants in the mid age range. King et al. [26] suggested this may be due to the “carefree” nature of younger generations who were perceived to be more willing to share their personal information (e.g. on social-networking sites) and older respondents who are no longer invested in their career and therefore under less scrutiny. More recently the Wellcome Trust [3] found a non-linear relationship between acceptance of commercial access to health data and age and noted that young people are not automatically more supportive/accepting. These varying and, at times, conflicting findings point to the need for greater research to explore the variations in perceptions and opinions across age groups.

Finally, the authors conducted a broad search of public responses to data sharing and data linkage in research that included studies looking at genetic data^97^ and medical-records data.^98^ These topics were considered together with other papers discussing health, personal or administrative data or information for statistical, health, social or other research purposes. Some studies suggest genetic data is particularly sensitive^99^ or personal/potentially identifying.^100^ In one study, participants perceived genetic data to be potentially less sensitive than information from medical records (e.g. information relating to reproductive or mental health).^101^ Participants’ from another study reported no real variation in attitudes toward the use of medical records and biological samples.^102^ In some studies, linking medical records data to biological samples raises concerns.^103^ However, overall opinions were largely consistent with the key themes of this review.

## Discussion

The included studies point towards widespread support for uses of data in research, including for practices of data-linkage and data-sharing. However, this support is never unconditional. Key conditions for public support or acceptance relate to the research being in “the public interest” or for “the greater good” and to public trust in researchers or organisations handling/accessing their data. The themes of public benefits and public trust run through all the studies (explicitly or implicitly) and underpin all other areas of concern or interest. As has been noted elsewhere [27] trust—or trustworthiness—is increasingly recognised as being central in shaping public responses. However, the included studies do not point to clear relationships or hierarchies between particular areas of concern or conditions for support and there is a lack of evidence relating to the ways in which trade offs might be made or how preferences would be formed in reality. This may represent a valuable area to explore further in future research.

As the literature in this area has frequently observed, confidentiality is a key area of public concern and assurances of confidentiality appear to be important for ensuring public support. However, in the wider literature relating to secondary uses of data in health research there has been much debate about the value and implications of anonymisation which is frequently described as representing significant challenges [28–30]. For example, it is argued that a certain amount of identifying information is needed in order to allow updating, linkage or validation of data [30, 31]. Ohm has argued that ‘data can either be useful or perfectly anonymous but never both’ [32] (p.1704). Despite these challenges relating to anonymisation, confidentiality is largely discussed and understood in terms of anonymisation. The included studies which explored public attitudes towards confidentiality typically focussed on attitudes towards anonymisation of data.

Anonymisation is generally understood as the process of removing key identifiers such as names and dates of birth from personal data thus rendering the identification of subjects highly unlikely. However, anonymisation is not straightforward and, as the MRC & Wellcome Trust suggest: ‘Because identifiability runs a spectrum, anonymisation is relative’ [2] (p.10). The UK Information Commissioner’s Office (ICO) has stated that ‘[i]n reality it can be difficult to determine whether data has been anonymised or is still personal data’ [33] (p.16). This ambiguity around anonymisation has implications for understanding public responses in this area, as Haddow et al. note, where studies have explored public attitudes ‘it is often unclear whether the research into publics’ views relates to fully anonymised data, the use of weaker forms of anonymisation or indeed fully identifiable data [34] (p. 1141). Therefore whilst studies have reported public attitudes towards anonymisation it is not always clear what members of the public understand anonymisation to mean, or what they perceive it to require.

There is evidence within the included studies that assurances of anonymisation may be important for members of the public, however those studies which enabled greater reflection on the implications or practicalities of anonymisation (e.g. through deliberative methods) typically uncovered more nuanced positions with members of the public often acknowledging that anonymisation is imperfect as a mechanism for protecting confidentiality and/or problematic for facilitating valuable research. Thus, anonymisation is not regarded as a panacea for addressing public concerns and it may be fruitful to explore further public attitudes towards confidentiality—and the ways that this might be ensured—beyond anonymisation of data.

Similarly, whilst the extant literature in this area has focussed heavily on the role and challenges of consent in relation to data-sharing or data-linkage for research purposes, the included studies highlight that this may not be a fundamental requirement for public acceptability. Rather, the studies indicate that whilst autonomy—or individual control over one’s data—is highly valued, consent is acknowledged to be problematic. As in discussions of anonymisation, where study participants have had opportunities to reflect on and discuss consent, views typically shift from an initial preference for explicit opt-in consent, towards more flexible models of either opt-out or varied consent. In some cases where study participants have been convinced of the value of research and the potential for public benefits consent has been regarded as non-essential. However, the degree of control individuals describe as necessary relates to the extent to which they trust the institutions, organisations or individuals involved in processing or accessing their data. A recent study conducted by Ipsos Mori on behalf of the Wellcome Trust found that whilst participants in their deliberative workshops initially tended to express preferences for opt-in consent models through the deliberative process, they shifted to a position where they “felt that if they knew more about the processes and safeguards in place they might feel more empowered, and hence more open and trusting in the decision-making process around data collection and sharing (and may not, therefore, need to opt-in)” [3] (p.13). Control may be facilitated through transparency and public engagement rather than direct or specific opt-in consent. As such, the findings reported in the included studies suggest that rather than focussing on which consent mechanisms are most favoured by members of the public, it may be more valuable to focus on how relationships of trust are built up (and conversely eroded) and how trust can be facilitated within research and data-sharing or data-linkage processes including through public/patient engagement or involvement.

This represents an important finding of this review. The literature has often suggested consent may be a requirement for public acceptability, whilst simultaneously arguing that requirements for consent present obstacles to effective and necessary health research and/or surveillance [29, 30, 35]. One alternative to consent which is currently used in the United Kingdom and elsewhere is authorisation. In England, for example, the Confidentiality Advisory Group (CAG) advises on requests to access data for research where neither consent nor anonymisation are deemed practicable. Similarly, the Public Benefit and Privacy Panel (PBPP) in Scotland is responsible for advising on data access requests involving personal data held by Information Services Division (ISD) of NHS National Services Scotland (NSS) and NRS (National Records of Scotland). Authorisation is now a widely used governance mechanism and authorising bodies play a significant role within the data sharing landscape. However, this review has found that to date the literature has not engaged with the subject of authorisation and there is a lack of evidence on public awareness of, or responses to, authorisation as a governance mechanism. The findings that individual level consent may not be crucial for public acceptance and that trust in organisations and institutions may be more important in shaping public responses, point to the salience of public engagement relating to authorisation approaches. Future research ought to explore public responses to authorisation.

As well as highlighting important conditions for public support, the included studies also indicate a number of areas of public concern about research uses of data. These relate largely to the purposes the research is perceived to serve, and the extent to which it is considered to be in the public interest or likely to yield public benefits. There is significant concern about potential misuse or abuse of data with negative implications for individuals, however there are also concerns about the potential for wider negative impacts from the outcomes of research. These relate to: the potential for data-sharing or data-linkage to enable, or perpetuate mass surveillance and a perceived “Big Brother Society”; the potential for individuals or groups within society to be labelled as a result of data-linkage research and for such labelling to result in stigma or discriminatory treatment, and to; the potential for research based on analysis of large data-sets to be used to inform policies or practices designed “for the masses” rather than reflecting individual circumstances and needs. What is apparent in relation to all these concerns is the underlying questioning of whether the research and its potential impacts/outcomes are perceived to be in the public interest or likely to bring about public benefits. The potential for research to lead to harm (directly or indirectly) is an area of significant concern.

The studies identified in this review reveal generally lower levels of trust in private sector actors compared with public sector actors alongside concern about private sector involvement in research. These concerns are often related to profit creation from use of individuals’ data and/or perceptions that data is routinely sold or passed on within the private sector. However, the studies do not suggest widespread opposition to private sector involvement, indeed many study participants acknowledged the important role of private sector actors in conducting or facilitating valuable research. Public support/acceptance of private sector involvement was largely conditional on the extent to which the research was perceived to be in the public interest or to lead to public benefits (as has recently been found by the Wellcome Trust [3]). Profit creation largely was not perceived as a problem so long as public benefits were prioritised over profits. The extent to which this was expected to be the case depended on the level of trust study participants had in the individuals or organisations handling/accessing data.

An important observation to emerge from this thematic synthesis is the public’s appetite for more information about current research and data-sharing or data-linkage practices. Many of the included studies reveal that there is generally very low public awareness of current research practices and governance systems or safeguards in place. There is evidence that those studies which used deliberative methods and provided participants with opportunities to learn more about current, or planned practices led to greater support/acceptance, or less concern about research uses of data. Additionally, almost all included studies reported that participants expressed a desire for more information and/or greater transparency about the ways in which data are used in research and the safeguards in place to protect against misuse/abuse or harms. This is significant and indicates not only that more awareness raising is needed but also that there may be significant enthusiasm amongst the public to engage more directly with and in these forms of research. Awareness raising should not be approached as a simple process of one-way information provision but rather requires a more engaged approach in order to ensure that it addresses public interests, concerns or uncertainties. The findings reported in the literature indicate that greater transparency may be needed, however, as we have previously noted, “research/researchers will be more likely to be perceived as trustworthy if transparency and public engagement involve open dialogue with members of the public and opportunities for deliberation, rather than controlled dissemination of information” [27] (p.9).

Within the included studies members of the public have been conceptualised in a number of ways. Some studies have suggested that uses of data in research—and particularly data-linkage—is a complex area which is difficult for members of the public to understand or meaningfully engage with. This leads to suggestions that awareness raising should be used to reassure members of the public through simple information provision and reflects a deficit model approach to public understanding of science [36].^104^ However, those studies which involved deliberative methods have demonstrated that members of the public were able and enthusiastic to engage in discussions on this subject and were competent and valuable deliberators.^105^ The nuanced positions described within the included studies highlight the value of qualitative methods for not only revealing but also informing and developing public attitudes. In this way qualitative methods themselves—as forms of public engagement—may have a role to play in building trust which in turn may underpin greater support for secondary uses of data. In this way increased use of qualitative methods might be a building block for support. Such public engagement and qualitative research are increasingly frequent components of large science projects and represent, in part, efforts to increase public trust and to ensure Responsible Research and Innovation (RRI) [27].

Overall this thematic synthesis has also revealed that there is great scope for qualitative methods to be used more fully or effectively in this area. This thematic synthesis has focussed only on qualitative studies—or qualitative findings reported within mixed methods studies—yet in some cases qualitative methods had been used primarily to inform the design of quantitative studies.^106^ Moreover, ten studies were excluded at the final stage due to their limited reporting of qualitative findings or their narrow, structured approach (e.g. qualitative methods being used to examine public responses to narrowly defined questions/hypotheses). Therefore it appears there may be a tendency for qualitative methods to be used largely as a means for informing subsequent quantitative methods, which in turn suggests an under-appreciation of the value of qualitative methods. Indeed, there is some evidence that qualitative methods may at times not be recognised as research methods. The authors found that only just over half of the included studies^107^ explicitly referred to ethical review procedures relating to the qualitative research while researchers in one study specifically stated that ethical approval was not required.^108^

### Study Limitations

Qualitative studies are sometimes criticised for their limited generalisability due to small and/or unrepresentative samples, such criticisms might be levied at the included studies within this thematic synthesis. The sample sizes ranged from 14 to 217 participants with the average being 54.84. Moreover, many of the included studies focussed on particular groups such as those with particular health conditions/susceptibilities,^109^ particular socio-demographic groups^110^ or with previous experience with research and/or data-sharing.^111^ Additionally, it is important to note that while random or quota sampling was often used^112^ the qualitative methods relied upon people volunteering to participate in the research which often involved a significant time commitment. Thus it might be speculated that those individuals who participated in these studies were more likely to be supportive of—or at least interested in—research and individuals who are less supportive, or more sceptical of research might have been less inclined to participate. Whilst these factors mean that the studies cannot be taken as being representative of the views of the wider public they remain valuable as indicators of the range of views within the public and particularly as illustrating how opinions are expressed and how they may be informed or influenced. This synthesis of the included studies has addressed some of the criticisms directed at qualitative studies in giving increased breadth through synthesising findings from a large (total) number of study participants and in a variety of contexts.

## Conclusion

With ever-growing interest in secondary uses of data for health research, including practices of data linkage and data sharing, there has increasingly been attention directed at public acceptability of these practices. Public acceptability is recognised as crucial for ensuring the legitimacy of current practices and systems of governance. This systematic review and thematic synthesis has highlighted a growing body of evidence pointing towards widespread general—though conditional—support for data linkage and data sharing for research purposes. It has found that whilst a variety of concerns are raised (e.g. relating to confidentiality, individuals’ control over their data, uses and abuses of data and potential harms arising) where members of the public perceive there to be actual or potential public benefits arising from research and where they have trust in the individuals or organisations conducting and/or overseeing data linkage/sharing they are generally supportive. However, the thematic synthesis has also highlighted current low levels of awareness about existing practices and uses of data, it points towards the need for greater awareness raising combined with opportunities for public engagement and deliberation. This will be important for ensuring the legitimacy of future health informatics research and for avoiding further public controversy.

## Appendix 1

### Adapted search strategy used in medline

1. (Lay or patient* or public or citizen$1).mp. [mp = title, abstract, original title, name of substance word, subject heading word, keyword heading word, protocol supplementary concept word, rare disease supplementary concept word, unique identifier]
2. exp Patients/
3. 1 or 2
4. (Attitude* or view$1 or perspective* or opinion$1).mp. [mp = title, abstract, original title, name of substance word, subject heading word, keyword heading word, protocol supplementary concept word, rare disease supplementary concept word, unique identifier]
5. exp attitude to health/ or health knowledge, attitudes, practice/
6. 4 or 5
7. 3 and 6
8. exp Public Opinion/
9. 7 or 8
10. (Record$1 or data).mp. [mp = title, abstract, original title, name of substance word, subject heading word, keyword heading word, protocol supplementary concept word, rare disease supplementary concept word, unique identifier]
11. (Share* or sharing or link$2 or linkage).mp. [mp = title, abstract, original title, name of substance word, subject heading word, keyword heading word, protocol supplementary concept word, rare disease supplementary concept word, unique identifier]
12. 10 and 11
13. exp Medical Record Linkage/
14. 12 or 13
15. Research.mp. [mp = title, abstract, original title, name of substance word, subject heading word, keyword heading word, protocol supplementary concept word, rare disease supplementary concept word, unique identifier]
16. *research/ or exp behavioral research/ or exp biomedical research/ or exp community-based participatory research/ or exp empirical research/ or exp human experimentation/ or exp operations research/ or exp parapsychology/ or exp peer review, research/ or exp research design/ or exp research report/ or exp social validity, research/
17. 15 or 16
18. (Access or purpose$1).mp. [mp = title, abstract, original title, name of substance word, subject heading word, keyword heading word, protocol supplementary concept word, rare disease supplementary concept word, unique identifier]
19. 17 and 18
20. 9 and 14 and 19
21. (qualitative or ethnograph* or “grounded theory” or “in depth interview$1” or “*structured interview$1” or “focus group$1” or “case study” or “case studies” or “case series” or “citizen$2 jury” or “citizen$2 juries” or vignette* or observation*).mp. [mp = title, abstract, original title, name of substance word, subject heading word, keyword heading word, protocol supplementary concept word, rare disease supplementary concept word, unique identifier]
22. exp qualitative research/
23. *Interview/
24. exp Focus Groups/
25. exp Observation/
26. 21 or 22 or 23 or 24 or 25
27. 20 and 26

## Footnotes

1 As indicated in Studies: 3, 7, 8, 10, 19, 21, 22, 25

2 Studies: 2, 3, 4, 7, 8, 9, 10, 12, 13, 14, 15, 18, 19, 20, 21, 22, 25

3 Studies: 7, 10, 11

4 Studies: 1, 2, 6, 19, 21, 22

5 Studies: 2, 3, 4, 5, 6, 9, 11, 14, 17, 18, 21, 22, 24

6 Studies: 1, 3, 5, 6, 9, 11, 14, 15, 17, 20, 21, 22

7 Study 5

8 e.g. Study 6

9 Studies: 8, 17

10 Studies: 1, 5, 8, 11, 14

11 Study 1

12 Studies: 1, 3, 11, 14

13 Studies: 1, 7, 8, 10, 11, 14, 18, 19, 21, 22, 24

14 Studies: 4, 5, 12, 14, 18, 22

15 Studies: 8, 10, 11, 14, 15, 17, 19, 20, 21, 25

16 Studies: 7, 10, 19, 21

17 Studies: 7, 8, 10, 11, 24

18 Studies: 1, 5, 6, 8, 11, 14, 20, 22, 24

19 Studies: 5, 8, 11

20 Studies: 5, 6, 8, 11, 23

21 Studies: 8, 10, 22

22 Studies: 8, 11, 19, 21, 22, 25

23 Studies: 1, 2, 4, 5, 6, 10, 11, 18, 21, 23, 25

24 Studies: 7, 11, 13, 19, 23

25 Study 5

26 Studies: 4, 5, 8, 9, 14, 15, 17, 21, 22, 24

27 Studies: 21, 22

28 Studies: 1, 2, 4, 5, 6, 10, 11, 18, 21, 23, 25

29 Studies: 4, 12, 14, 18, 23, 25

30 Studies: 2, 12, 14, 18, 23, 25

31 Studies: 4, 14, 15, 17, 25

32 Studies: 4, 25

33 Studies: 5, 10, 12, 18, 25

34 Studies: 12, 15, 23, 25

35 Studies: 2, 11, 12, 14, 20, 22, 23, 24

36 Studies: 14, 15, 19, 23

37 Studies: 10, 19

38 Studies: 4, 5, 12,18, 22, 23, 24

39 Studies: 16, 23, 24

40 Studies: 11, 15, 16, 20, 21, 23, 25

41 Studies: 8, 10, 12, 17, 21, 22, 23

42 Studies: 5, 20, 21

43 Study: 3

44 Studies: 18, 19, 24

45 Study: 18

46 Studies: 8, 17, 20, 23, 25

47 Studies: 4, 5, 8, 10, 11, 12, 13, 14, 21, 22

48 Studies: 4, 8, 9, 11, 12, 14, 19, 21, 22, 23, 25

49 Studies: 5, 11, 21, 24

50 Studies: 5, 9, 11, 12, 13, 19, 21, 22, 23

51 Study: 14

52 Studies: 17, 21, 23

53 Studies: 5, 11, 13, 14, 22

54 Studies: 1, 5, 6, 8, 11, 14, 19

55 Studies: 1, 5, 6, 9, 11, 12, 17, 24

56 Studies: 6, 7, 11, 15, 16, 17, 20, 22, 23

57 Studies: 5, 6, 7, 8, 9, 10, 11, 12, 14, 17, 20, 22, 24

58 Studies: 4, 5, 10, 11, 12, 14, 18, 21, 22, 23, 25

59 Studies: 6, 10,11,20,22

60 Studies: 6, 7, 10, 11, 21, 22, 25

61 Studies: 3, 22

62 Studies: 6, 10, 14, 18, 21, 25

63 Study: 18

64 Study: 6

65 Studies: 6, 11, 22

66 Studies: 17, 25

67 Studies: 8, 11, 21, 22

68 Studies: 6, 7, 8

69 Studies: 6, 8

70 Study: 15

71 Studies: 5, 10, 15, 21, 25

72 Studies: 6, 11, 15

73 Studies: 9, 11, 12, 22, 24

74 Studies: 7, 11, 12, 20, 22

75 Study: 4

76 Studies: 2, 4, 6, 11, 14, 22

77 Studies: 2, 4, 6, 8, 9, 11, 13, 14, 21

78 Studies: 6, 11, 21

79 Studies: 5, 7, 8, 14, 15, 17, 25

80 Studies: 4, 17, 20, 22, 24, 25

81 Studies: 5, 6, 11

82 Studies: 5, 14, 15

83 Study: 17

84 Studies: 2, 4, 5, 6, 7, 9, 10, 11, 12, 13, 14, 15, 19, 21, 23, 24

85 Studies: 12, 14, 18, 21

86 Studies: 6, 7, 11, 14, 15, 18, 19

87 Studies: 5, 6, 11

88 Studies: 5, 6, 15

89 Study: 2

90 Study: 4

91 Study: 9

92 Study: 6

93 Studies: 22, 23

94 Study: 14

95 Study: 23

96 Study: 22

97 Studies: 2, 9, 13, 19, 22, 23

98 Studies: 2, 4, 10, 15, 16, 17, 20, 24, 25

99 Studies: 13, 14

100 Studies: 2, 13, 18, 11, 12, 24

101 Study: 22

102 Study: 2

103 Studies: 18, 9, 22

104 Such a position is implicit in Studies:, 7, 10, 11, 14, 15, 16, 17, 19

105 Studies: 4, 5, 6, 18

106 Studies: 4, 13, 24, 25

107 Studies: 3, 4, 9, 10, 12, 13,15, 17, 19, 20, 22, 23, 24, 25

108 Study 17

109 Studies: 3, 8, 12, 14, 16

110 Studies: 2, 4, 6, 8, 9, 10, 17, 19, 24

111 Studies: 12, 13, 22, 23, 24

112 Studies: 4, 5, 6, 7, 10, 11, 14, 20, 22, 23

## References

[1] WMA. *WMA Declaration on Ethical Considerations Regarding Health Databases*. WMA; 2002. http://www.wma.net/en/30publications/10policies/d1/(accessed 9th June 2016)

[2] MRC & Wellcome Trust. *Access to Collections of Data and Materials for Health Research*. Wellcome Trust; 2006. www.wellcome.ac.uk. Accessed 03 Nov 2016.

[3] Wellcome Trust (2016). The One-Way Mirror: Public attitudes to commercial access to health data.

[4] Bradwell & Gallagher. *FYI: The new politics of personal information*. 2007. www.demos.co.uk. Accessed 03 Nov 2016.

[5] Robling MR, Hood K, Houston H, Pill R, Fay J, Evans HM (2004). Public attitudes towards the use of primary care patient record data in medical research without consent: a qualitative study. J Med Ethics.

[6] Barbour RS, Barbour M (2003). Evaluating and synthesising qualitative research: the need to develop a distinctive approach. J Eval Clin Pract.

[7] Pope C, Ziebland S, Mays N (2000). Qualitative research in health care: Analysing qualitative data. Br Med J.

[8] Popay J, Rogers A, Williams G (1998). Standards for the systematic review of qualitative literature in health services research. Qual Health Res.

[9] Davidson S, McLean C, Treanor S, Aitken M, Cunningham-Burley S, Laurie G, Sethi N, Pagliari C (2013). Public acceptability of data sharing between the public, private and third sectors for research purposes. (Social Research series).

[10] Wellcome Trust, *Enabling data linkage to maximise the value of public health research data: full report* Wellcome Trust; 2015 www.wellcome.ac.uk. Accessed 03 Nov 2016.

[11] Holman CDJ, Bass AJ, Rosman DL, Smith MB, Semmens JB, Glasson EJ, Brook EL, Trutwein B, Rouse IL, Watson CR, de Klerk NH, Stanley FJ (2008). A decade of data linkage in Western Australia: strategic design, applications and benefits of the WA data linkage system. Aust Health Rev.

[12] Young TK, Kliewer E, Blanchard J, Mayer T (2000). Monitoring disease burden and preventive behavior with data linkage: cervical cancer among aboriginal people in Manitoba, Canada. Am J Public Health.

[13] Fischbacher CM, Bhopal R, Povey C, Steiner M, Chalmers J, Mueller G, Jamieson J, Knowles D (2007). Record linked retrospective cohort study of 4.6 million people exploring ethnic variations in disease: myocardial infarction in South Asians. BMC Public Health.

[14] Veugelers PJ, Yip AM, Kephart G (2001). Proximate and contextual socioeconomic determinants of mortality: multilevel approaches in a setting with universal health care coverage. Am J Epidemiol.

[15] Jutte DP, Roos LL, Brownell MD (2011). Administrative record linkage as a tool for public health research. Annu Rev Public Health.

[16] Kariminia A, Butler TG, Corben SP, Levy MH, Grant L, Kaldor JM, Law MG (2007). Extreme cause-specific mortality in a cohort of adult prisoners—1988 to 2002: a data-linkage study. Int J Epidemiol.

[17] CASP (2013). CASP Qualitative Checklist.

[18] Thomas J, Harden A (2008). Methods for the thematic synthesis of qualitative research in systematic reviews. BMC Med Res Methodol.

[19] Britten N, Campbell R, Pope C, Donovan J, Morgan M, Pill R (2002). Using meta-ethnography to synthesise qualitative research: A worked example. J Health Serv Res Policy.

[20] Ishikawa H, Yamazaki Y (2005). How applicable are western models of patient-physician relationship in Asia?: Changing patient-physician relationship in contemporary Japan. Int J Jpn Sociol.

[21] Pentz RD, Billot L, Wendler D (2006). Research on stored biological samples: Views of African American and White American cancer patients. Am J Med Genet.

[22] Mezuk B, Eaton WW, Zandi P (2008). Participant characteristics that influence consent for genetic research in a population-based survey: the Baltimore epidemiological catchment area follow-up. Community Genet.

[23] Hartz SM, Johnson EO, Saccone NL, Hatsukami D, Breslau N, Bierut LJ (2010). Inclusion of African Americans in genetic studies: what is the barrier?. Am J Epidemiol.

[24] Devers K, Gray B, Ramos C, Shah A, Blavin F, Waidmann T. The feasibility of using electronic health records (EHRs) and other electronic health data for research on small populations. Washington: Urban Institute; 2013.

[25] Buckley BS, Murphy AW, MacFarlane AE (2011). Public attitudes to the use in research of personal health information from general practitioners’ records a survey of the Irish general public. J Med Ethics.

[26] King T, Brankovic L, Gillard P (2012). Perspectives of Australian adults about protecting the privacy of their information in statistical databases. Int J Med Inform.

[27] Aitken, M., Cunningham-Burley, S., & Pagliari, C. Moving from trust to trustworthiness: Experiences of public engagement in the Scottish Health Informatics Programme. *Sci Public Policy.* 2016. doi: 10.1093/scipol/scv075. 10.1093/scipol/scv075 PMC5210028 28066123

[28] Campbell B, Thomson H, Slater J, Coward C, Wyatt K, Sweeney K (2007). Extracting information from hospital records: what patients think about consent. Qual Safety Health Care.

[29] Chalmers J, Muir R (2003). Patient Privacy and Confidentiality: The debate goes on; the issues are complex but a consensus is emerging. Br Med J.

[30] Verity C, Nicoll A (2002). Consent, confidentiality and the threat to public health surveillance. Br Med J.

[31] Strobl J, Cave E, Walley T (2000). Data Protection Legislation: Interpretation and barriers to research. Br Med J.

[32] Ohm P (2010). Broken promises of privacy: responding to the surprising failure of anonymity. UCLA Law Rev.

[33] ICO. Anonymisation: managing data protection risk code of practice. Cheshire: Information Commissioner’s Office (ICO); 2012.

[34] Haddow G, Bruce A, Sathanandam S, Wyatt JC (2011). “Nothing is really safe”: a focus group study on the processes of anonymizing and sharing of health data for research purposes. J Eval Clin Pract.

[35] Damschroder LJ, Pritts JL, Neblo MA, Kalarickal RJ, Creswell JW, Hayward RA (2007). Patients, privacy and trust: Patients’ willingness to allow researchers to access their medical records. Soc Sci Med.

[36] Gross AG (1994). The roles of rhetoric in the public understanding of science. Public Underst Sci.

[37] Booth, A.*Cochrane or cock-eyed? How should we conduct systematic reviews of qualitative research?* 2001; http://www.leeds.ac.uk/educol/documents/00001724.htm. Accessed 03 Nov 2016.

[38] Sandelowski M, Barroso J (2003). Classifying the findings in qualitative studies. Qual Health Res.

